# Consequences of Cancer on Zebrafish *Danio rerio*: Insights Into Sex Determination, Sex Ratio, and Offspring Survival

**DOI:** 10.1002/ece3.72003

**Published:** 2025-08-31

**Authors:** Justine Boutry, Mathieu Douhard, Klara Asselin, Antoine M. Dujon, Jordan Meliani, Olivier De Backer, Delphine Nicolas, Aaron G. Schultz, Peter A. Biro, Christa Beckmann, Laura Fontenille, Karima Kissa, Beata Ujvari, Frédéric Thomas

**Affiliations:** ^1^ CREEC/CANECEV (CREES), MIVEGEC, Unité Mixte de Recherches IRD 224–CNRS 5290–Université de Montpellier Montpellier France; ^2^ Laboratoire de Biométrie et Biologie Evolutive UMR 5558 Université de Lyon, Université Lyon 1, CNRS Villeurbanne France; ^3^ School of Life and Environmental Sciences Deakin University Waurn Ponds Victoria Australia; ^4^ Unité de Recherche en Physiologie Moléculaire (URPhyM), Namur Research Institute for Life Sciences (NARILIS) University of Namur Namur Belgium; ^5^ Tour du Valat, Institut de Recherche Pour la Conservation Des Zones Humides Méditerranéennes Arles France; ^6^ School of Science RMIT University Melbourne Victoria Australia; ^7^ AZELEAD Montpellier France; ^8^ University of Montpellier, VBIC, INSERM U1047 Montpellier France

**Keywords:** cancer ecology, *Danio rerio*, maternal effects, offspring sex ratio, reproductive investment

## Abstract

Offspring sex ratio has been proposed as an indicator of the risk of developing certain cancers in humans, but offspring sex ratio may also be a consequence of the disease. In this study, we investigate this subject using the zebrafish, *Danio rerio,* as a model system. First, we explore whether inducing skin cancer at an early stage of the host's life (embryonic stage) has the potential to influence sex determination and/or sex‐specific mortality. Second, we investigate whether the sex ratio in offspring produced by tumor‐bearing adult females differs from that of healthy females. Third, we compare the survival (until sexual maturity) of offspring produced by cancerous and non‐cancerous females. We found that skin cancer did not influence sex determination and the sex ratio of the offspring. However, consistent with previous studies on other model systems, the survival of offspring was higher when mothers were cancerous, suggesting that diseased females allocate more resources to current reproductive effort compared to their healthy counterparts. This study makes a significant contribution to our understanding of the ecological and evolutionary consequences of host‐tumor interactions in animals.

## Graphical abstract

We investigated how cancer influences reproductive traits in zebrafish (*Danio rerio*), focusing on sex determination, offspring sex ratio, and juvenile survival. While embryonic cancer induction did not affect sex ratios, we found that tumor‐bearing females produced more offspring with higher survival rates, suggesting enhanced maternal investment under disease. These findings provide new insights into the ecological and evolutionary consequences of cancer in animal hosts.

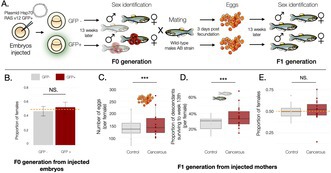

## 1 Introduction

While it is now widely acknowledged that numerous human activities promote cancerous pathologies in wildlife (Baines et al. 2021; Giraudeau et al. 2018; Sepp et al. 2019), the exact magnitude of the resulting ecological and evolutionary consequences remains incompletely understood (Thomas et al. 2017; Vittecoq et al. 2013). Simply predicting the accelerated disappearance of animals that have developed tumors in ecosystems is an oversimplified viewpoint that overlooks other potential repercussions. For instance, at the ecosystem level, the ecological impacts of differing susceptibility to cancer among species will vary based on the functional traits of the most affected species (e.g., predators, prey, keystone species, Hamede et al. 2020; Perret et al. 2020). Phenotypic changes induced by oncogenic processes in hosts also have the potential to alter various interactions between affected organisms and other species within the ecosystem. For example, tumor‐bearing hydra exhibit modified interactions with other species compared to their healthy counterparts—they capture more prey, are more susceptible to predation, and experience heavier colonization by commensal ciliates (Boutry, Mistral, et al. 2022; Duneau et al. 2011). At both the individual and species levels, Dujon et al. (2024) suggested that the effects of cancer on life‐history traits should be considered across different timescales, particularly in the context of both sudden and chronic exposure to mutagenic substances. Initially, it is expected that organisms will over‐activate their anticancer defenses such as apoptosis, cellular senescence, DNA repair pathways, antioxidant systems, immune surveillance mechanisms (including natural killer cells and cytotoxic T lymphocytes), and tumor suppressor gene activation like p53, significantly impacting them because these defenses are energetically costly and may force energetic trade‐offs (Biro et al. 2020; Dujon et al. 2022; Klaassen et al. 2024; Thomas et al. 2019). It is also anticipated that affected species may progressively alter their life‐history traits, for example favoring earlier investment in reproduction (Arnal et al. 2017; Boutry, Tissot, et al. 2022; Jones et al. 2008). In the long term, natural selection may favor the development of more powerful anticancer defenses, such as duplications of tumor suppressor genes (Sulak et al. 2016; Trivedi et al. 2023). Thus, the consequences of anthropogenically induced oncogenic activities on wildlife are diverse and complex, warranting further in‐depth research, especially in aquatic ecosystems that are particularly prone to pollution (Häder et al. 2020).

Cancer has been documented in some aquatic vertebrate species, providing valuable insights into its potential ecological and evolutionary implications. For example, *Xiphophorus cortezi* , a species of freshwater fish, carries a cancer‐causing gene that is associated with traits such as male aggression and body size, both of which can have significant reproductive consequences. These findings illustrate the complex interactions between cancer and life‐history traits in shaping reproductive strategies (e.g., Fernandez 2010; Fernandez and Morris 2008), and emphasize the importance of investigating cancer's role in wildlife populations to better understand its broader ecological effects. The zebrafish ( *Danio rerio* ) is a widely used model for studying cancer due to its ability to develop cancer after exposure to mutagens or gene manipulation (Bambino and Chu 2017). Aquatic ecosystems are particularly vulnerable to pollution, and it is known that habitat pollution is linked to an increase in cancer risk (Baines et al. 2021). However, cancer incidence in natural populations, including zebrafish, remains largely unknown due to significant practical challenges. Diagnosing cancer in wildlife is particularly difficult because non‐invasive diagnostic methods are limited, obtaining sufficient well‐preserved specimens for autopsy is rare, and weakened animals often fall prey to predators, causing their bodies to disappear before examination (Hamede et al. 2020). Although studying cancer in natural populations is crucial to understanding its ecological consequences, this remains logistically and ethically challenging. In this context, laboratory models offer key advantages: they enable fine‐scale control over genetic and environmental variables, allow replication, and facilitate the use of mechanistic tools such as transgenesis. While not natural or wild, such models provide valuable insights and case studies into how cancer can affect life‐history traits and reproductive strategies under ecologically relevant constraints. Many tumors that *Danio* develop are highly homologous with human cancers at histological, proteomic, and genetic levels (Kobar et al. 2021). In addition, a mixture of organic pollutants common in today's environments can affect the development and behavior of zebrafish, highlighting the potential threats pollutants represent for human and animal populations (Bambino and Chu 2017). However, the extent to which the higher incidence of tumors could have population‐wide repercussions through changes in life‐history traits (Boddy et al. 2015; Ujvari et al. 2016) such as sex ratios or reproductive investment, has so far received little consideration. In this study, we chose to focus on skin cancer for its ecological relevance, but also its experimental tractability. Indeed, our use of the H‐RASV12 zebrafish model provides a validated and reproducible method to induce neoplasia (Santoriello et al. 2009), avoiding the variability and ethical concerns of environmental carcinogens. This model allows controlled tumor onset during early development, precisely when sex differentiation occurs, making it ideally suited to test the impact of tumor presence on sex ratio and reproductive investment.

Cancer is known to affect reproduction in various species, sometimes altering reproductive modes, as observed in hydra where cancer has been linked to shifts between sexual and asexual reproduction (Boutry, Tissot, et al. 2022). Such examples highlight the potential for oncogenic processes to influence reproductive traits across taxa, making it critical to explore these dynamics in other model systems. While the proximal mechanisms of sex‐ratio bias can rely on different mechanisms and physiological disruptions, rather than cancer itself directly affecting sex allocation, it is worth exploring the ultimate evolutionary consequences of such disturbances. These may include potentially adaptive shifts in sex allocation, favoring the sex less affected by cancer or more successful in reproduction under the constraints of disease development.

The zebrafish is a fascinating model organism to study sex allocation and reproduction trade‐offs, due to their multifactorial sex determination mechanisms. All zebrafish larvae develop ovary‐like tissue between 13 and 25 days post fertilization (Kossack and Draper 2019). Between 20 and 25 days post‐fertilization, the early‐stage oocytes of individuals that were determined as female continue to mature, while in individuals that were determined as male, early‐stage oocytes undergo apoptosis during the gonadal transition from a proto‐ovary into a functional testis. Sexual maturation is not complete until zebrafish are 2.5–3 months old (Kimmel et al. 1995). However, the times listed above are approximative and can vary depending on rearing conditions such as temperature (Santos et al. 2017). The mechanism of sex determination in zebrafish is complex, but once determined, sex remains the same throughout life. While wild zebrafish have a genetic WZ system (Wilson et al. 2014), this sex determinant appears to have been modified during domestication (Kossack and Draper 2019). In domesticated lines, a polygenic system regulates sex, but the particular genes involved vary between the independently selected lines. It is also known that sex ratios in domesticated lines vary depending on environmental factors, such as temperature, pH, oxygen concentration, diet, food availability, and rearing density (Kossack and Draper 2019). However, the extent to which cancer can act as a factor influencing sex determination has never been addressed to our knowledge.

The aim of this study was to provide one of the first experimental studies investigating the effects of cancer on biological traits relevant to evolutionary ecology. First, we examine the possibility that skin cancer occurring at a very early age of the host (i.e., embryonic) could affect sex‐specific mortality and/or sex determination, which is partially influenced by the environment in zebrafish (Experiment 1). This bias may occur due to a higher mortality among male embryos developing cancer compared to female embryos (Lary and Paulozzi 2001; Li et al. 2017; Poulin 1996; Wells 2000). Because sex differences in juvenile mortality may influence the strength of selection for sex allocation (Cox and Calsbeek 2010), cancer may increase the likelihood of producing females rather than males. Next, in a distinct investigation, we examined whether adult females bearing tumors, compared to healthy females, exhibit a sex ratio bias in their brood (Experiment 2). This hypothesis was motivated in part by the influence of toxic substances or high population density on the sex ratio of subsequent generations through epigenetic mechanisms in zebrafish (Guirandy et al. 2022; Pierron et al. 2021). A biased sex ratio favoring males could also occur due to the expectation that females with health issues will produce lower‐quality eggs. Regarding the overall survival of offspring in Experiment 2, a first potential prediction is that, being the progeny of mothers in poor health, these offspring start life with a disadvantage, potentially reducing their chances of survival. On the other hand, it cannot be dismissed that females with cancer, as observed in other species, allocate more resources to their immediate reproductive events (Guirandy et al. 2022; Pierron et al. 2021). This, as a terminal investment in reproduction (Clutton‐Brock 1984), may contribute to the enhanced survival of their offspring. To summarize, this study investigates three main hypotheses about how cancer can impact reproductive traits in zebrafish:

Hypothesis 1 *Early‐onset skin cancer may influence sex‐specific mortality and sex ratio determination. Male embryos are predicted to experience higher mortality rates than females, potentially leading to an altered sex ratio* (*Experiment* 1).

Hypothesis 2 *Tumor‐bearing females may exhibit changes in reproductive investment strategies, potentially resulting in a biased sex ratio in their broods compared to healthy females* (*Experiment* 2).

Hypothesis 3 *Cancer progression in females may affect maternal investment, influencing offspring survival. Offspring from cancerous females are predicted to exhibit higher survival rates due to increased maternal investment* (*Experiment* 2).

## 2 Material and Methods

### 2.1 Rearing Protocol

Zebrafish were hatched, raised, and monitored by AZELEAD fish facility (Montpellier, France). All experimental procedures on zebrafish were carried out in accordance with European directives and French Ministry of Health animal protection regulations (approval number: F341725). The experiments were approved by the ethics committee for animal experimentation (Direction départementale de la protection des populations—Hérault). The study was conducted in accordance with ARRIVE guidelines.

Fish larvae from their 4th to 10th day were raised in glass Petri dishes of 10 cm diameter with a maximal density of 1 embryo/cm^2^ and fed dry food (75 μg, *Plantkovie, France*) four times daily. Juvenile fish between their 10th and 75th day post fertilization were raised in 1 L (22.4 × 11.1 × 8.4 cm) tanks at a maximal density of 50 fish and fed with a mixture of Artemia nauplii (*Artemia salina, JBL, Germany*) twice daily and dry food (200 μg) four times a day. Adult fish older than 75 days were raised in groups of 15 to 33 individuals in 2.8 L (33.5 × 11.4 × 15.2 cm) tanks and fed with both Artemia nauplii *(Artemia salina, JBL)* and dry food (300 μg) twice daily. A total of 377 adult individuals were used in total in 17 different tanks in the first experiment. In the second experiment, females were held individually in 1 L tanks. *Artemia* nauplii were hatched in oxygenated salt water (36 g/L of NaCl). All fish (including larvae and juveniles) were maintained in a standard fish water medium (pH 7, 700 μS/cm, containing 150 mg/L CaCO_3_ and 60 mg/L NaCl) at 28°C with a 12 h/12 h dark/light cycle.

### 2.2 Breeding Procedure

The breeding tanks were maintained at 28°C with a 12‐h light/12‐h dark cycle, as described in the rearing protocol. The addition of glass marbles was used to mimic natural spawning conditions and prevent predation by adults, while dividers allowed for controlled mating interactions. All procedures were conducted in accordance with ethical guidelines. Embryos were collected at the end of the day (6:00 PM) rinsed with reverse osmosis water, and transferred to a Petri dish filled with standard breeding water at 28°C.

### 2.3 F0 Reproduction Monitoring—Experiment 1

**FIGURE 1:**
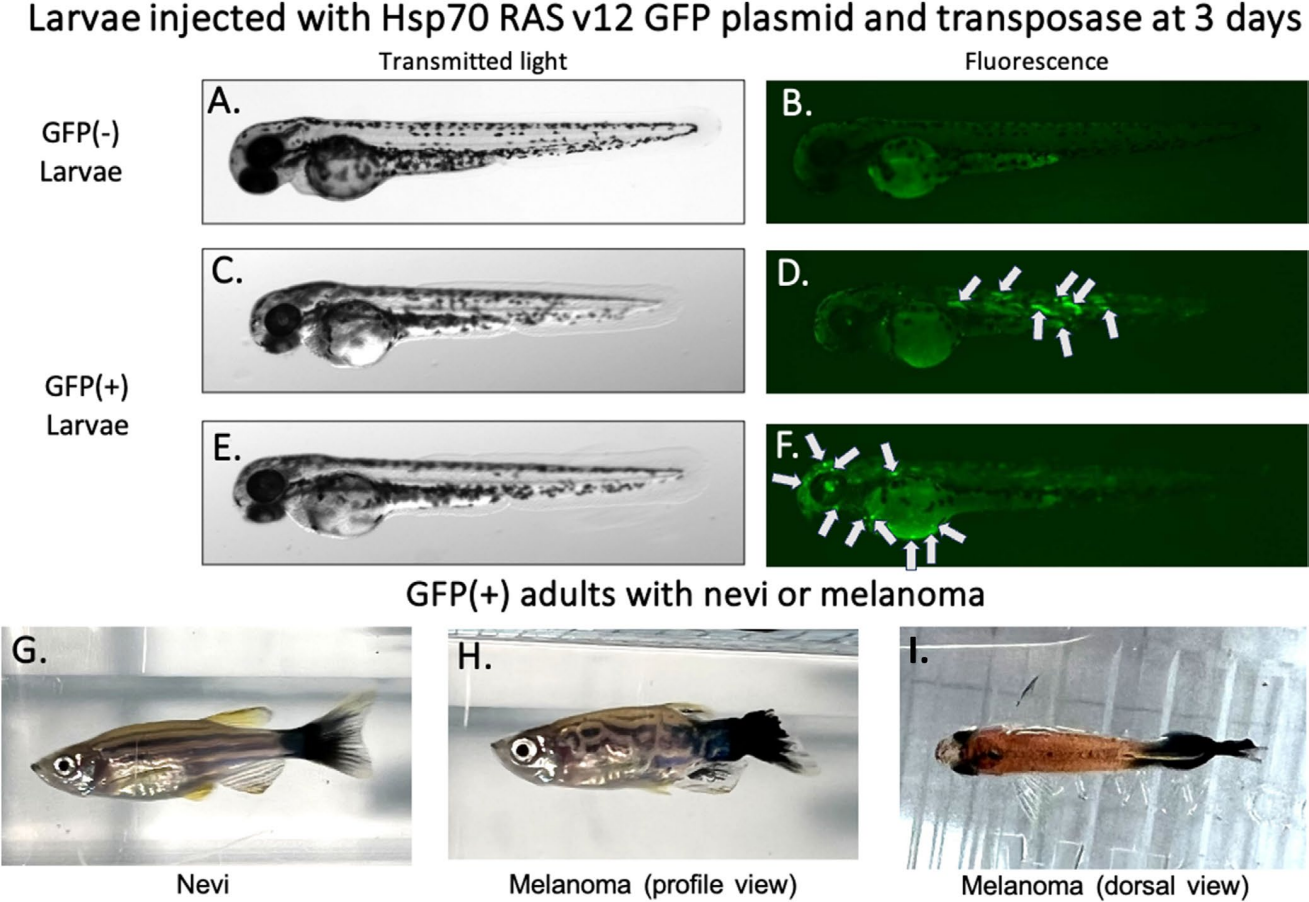
Pictures of larvae (A to F) and adult fish (G to I) injected with the oncogenic HSp70RASv12 and transposase plasmid. (A and B) depict larvae (3 days post‐injection) under transmitted light and fluorescence, respectively, that did not incorporate the plasmid and are consequently GFP‐negative. (C to F) represent pictures through transmitted light and fluorescence of two larvae (3 days post‐injection) that have integrated the fluorescent plasmid and are thus GFP‐positive (fluorescent areas are indicated by arrows). After 13 weeks of follow up, it is possible to distinguish in the adult fish population some individuals that do not show nevi manifestation (G) and melanoma (H and I). (A–F) Lateral view, I. Dorsal view.

All individuals used in this study belong to a wild type strain named AB line (Brown et al. 2015; Stuart et al. 1990). Embryos from the F0 were collected in the morning at stage 1 from 4 different siblings (2 males and 2 females). Fertilized embryos (*N* = 377) were injected with 1 nL of a mixture containing 25 ng/μL of the circular plasmid T2KSAG:GFP‐H‐RASV12 (hereafter abbreviated GFP) and 25 ng/μL of T2 transposase mRNA, as described in Santoriello et al. (2009). A heat shock was applied at 38°C 24 h post‐injection to activate plasmid expression. Injected embryos were hatched in plastic Petri dishes (see above) and their fluorescence was observed at 48 h post‐fertilization to identify and select embryos expressing the H‐RASV12 oncogene (see Figure 1). Fluorescence observations were performed using a Zeiss SteREO Discovery V8 stereomicroscope equipped with an Axiocam camera and an LED fluorescence illumination system optimized for GFP detection (excitation 470/40 nm, emission 525/50 nm). Accordingly, only GFP+ individuals exhibiting visible signs of cancer and GFP– individuals showing no detectable abnormalities were included in the cancerous and control groups, respectively. This design controls for the injection procedure and accounts for potential mosaicism, allowing us to isolate the specific effects of oncogene expression in tumor‐bearing individuals. The level of mosaicism in plasmid expression was not quantified in this study, which may have implications for interpreting the results. From 4 to 10 days post‐fertilization, larvae were reared in 10 cm glass Petri dishes at 28°C (1 embryo/cm^2^) and fed four times daily with dry food (75 μg, Plantkovie, France), with excess food removed using a glass pipette 30 min later and the medium was changed twice per day to avoid water pollution. After 10 days, larvae and juveniles were transferred to 1 L plastic tanks in the main system, with a maximal density of 50 individuals per tank. Sex identification and tumor presence were checked at 13 weeks based on visual screening (see Figure 1). As previously described (Santoriello et al. 2009), 20% of injected fish with the oncogenic H‐RASV12 developed neoplasia, mainly nevi, melanomas, and lymphomas; then the emergence of tumors on cancerous fish was observed daily using the method described in Tissot et al. (2023). We note that, in the early stages of the experiment, a PBS‐injected control group was included but later discarded due to its lack of significant effects on target traits. Also, some non‐injected individuals with visible developmental abnormalities were not retained for analysis, although their exact proportion was not recorded. This precaution was taken to maintain group homogeneity.

### 2.4 F1 Reproduction Monitoring—Experiment 2

**FIGURE 2:**
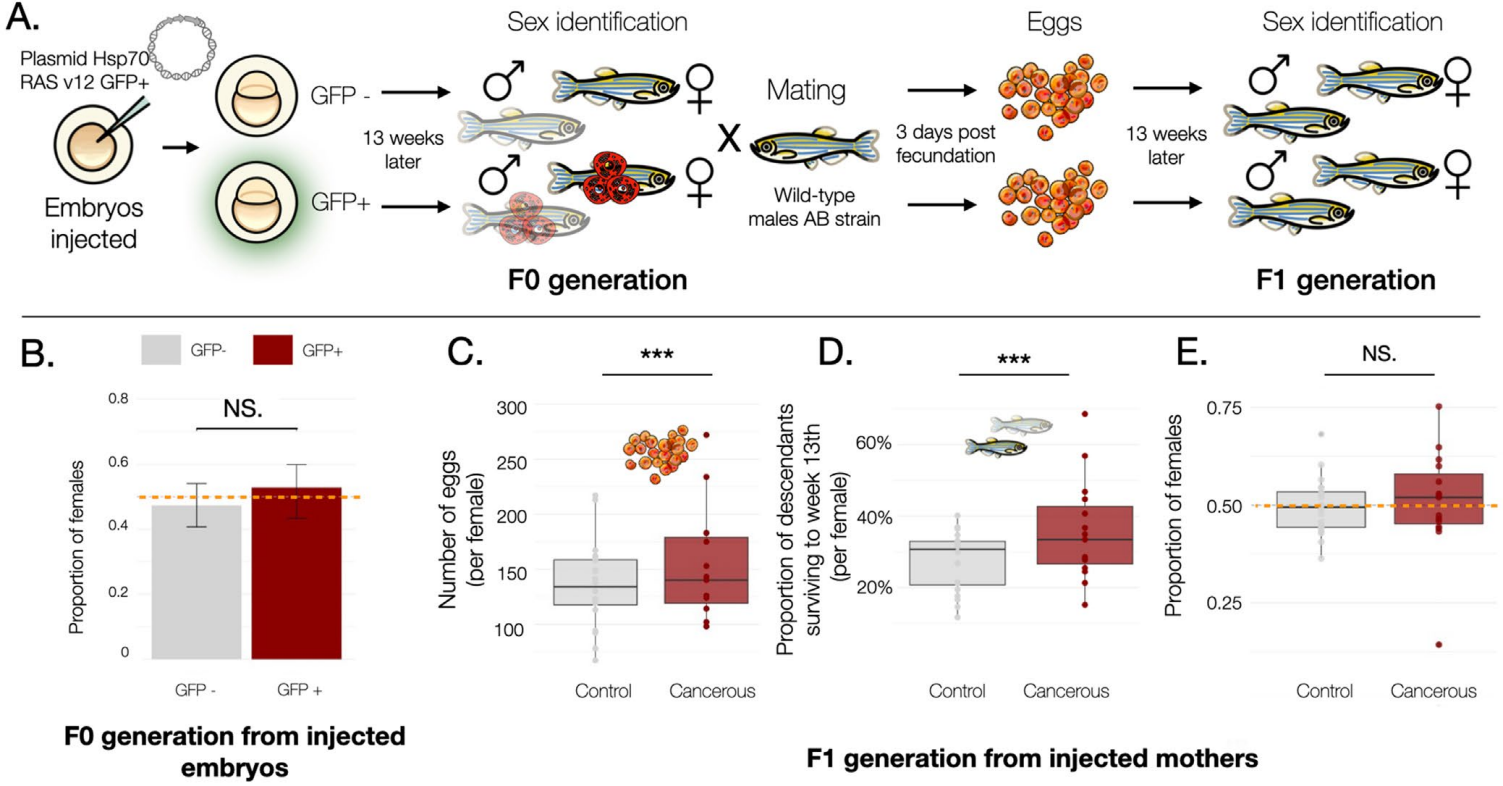
Monitoring the sex ratio of two successive generations after inducing skin cancer in zebafish ( *Danio rerio* ). (A) Experimental design: Embryos were injected with a oncogenic plasmid. After 48 h, integration and expression of the plasmid was screened through GFP fluorescence. Skin cancer was only observed among the GFP+ group. The individuals emerging from those embryos (F0) were sorted by sex at the 13th week and females were mated with wild‐type males. The number of embryos obtained from the female fish was counted 4 days after fecundation, and the sex of the individuals emerging from those embryos (F1) was identified at the 13th week. (B) The proportion of female fish in the F0 generation injected with an oncogenic plasmid was determined based on whether the plasmid was expressed (GFP+ group in red on the right, *n* = 228) or not (GFP‐ group in gray on the left, *n* = 149). There was no statistically significant difference (N.S) in the proportion of females between the two groups as the best selected binomial model was the null model (see Table S1). (C) The number of embryos 4 days after fecundation in the F1 generation of fish was recorded based on whether the mother was cancerous (positive group in red on the right, *n* = 15) or not (negative group in gray on the left, *n* = 20). There was a statistically significant increase in the number of embryos for tumorous females (Poisson glm, estimate = 0.23213, Incidence rate ratio (IRR) = 1.26 [1.18–1.34], *p* = 7.63e‐13***, see Table S2). (D) Average proportion per female of descendants in the F1 generation still alive at the 13th week, categorized by whether the mother was cancerous (positive group in red on the right, *n* = 15) or not (negative group in gray on the left, *n* = 20). There was a statistically significant increase in the proportion of descendants surviving to the 13th week for tumorous females (Binomial glm, estimate = 0.17437, Odd ratio (OR) = 1.19 [1.05–1.35], *p* = 0.00512***, see Table S3). (E) Proportion of female F1 fish generation based on whether the mother was cancerous (GFP+ group in red on the right, *n* = 15) or not (GFP‐ group in gray on the left, *n* = 20). There was no statistically significant (N.S) difference in the proportion of females between the two groups as the best selected binomial model was the null model (see Table S4). *** indicates *p* < 0.01; NS. indicates not significant.

Cancerous females were isolated from the F0 generation and mated with wild‐type males from the AB strain to generate an F1 generation. Fish husbandry was as described above. The total number of embryos produced and the sex of each F1 fish were observed at week 13. The complete experimental design is represented in Figure 2A.

### 2.5 Statistical Analysis

The sex ratios within the F0 and F1 generations were compared employing binomial distribution with Generalized Linear Mixed Models (GLMMs). To identify the most pertinent models, we undertook a three‐selection process based on the corrected Akaike Information Criterion (AICc) and associated weights (Zuur et al. 2009). First, models were compared incorporating potential combinations of random effects, including the common aquarium, experimental batch for the F0, the breeding date, and the experimental batch for the F1. Subsequently, models were evaluated considering the fixed effects of plasmid injection (given that it is the main effect we want to test), as well as the number of embryos and the number of mature adults in the tank at the 13th week for the F0. Sex ratio in the F0 and F1 generations and the proportion of embryos developed from the eggs hatched by the F0 females were modeled using binomial distribution as the response variable is binary (i.e., male or female, dead or alive). The number of embryos produced by the F0 females was modeled using a Poisson distribution as the response variable is a count. Analyses were conducted using R (R version 4.3.1) through the interface Rstudio (Version 2023.12.0 + 369). Data files were imported using the readr package (Wickham et al. 2023), and graphical exploration of the data was conducted using GGally (Schloerke et al. 2021). Statistical models were primarily fitted using the glmmTMB package (Brooks et al. 2017) for GLMMs. Model selection criteria were applied with the MuMIn package (Bartoń 2023) used for AIC calculation. The equilibrium and dispersion of residuals were evaluated using the DHARMa package (Xie et al. 2021), ensuring the robustness and appropriateness of the selected models. Additional analyses were carried out using the MASS package (Venables and Ripley 2002) for supplementary statistical functions. Results were visually presented using the sjPlot package (Lüdecke 2023) and graphs were generated using ggplot2 (Schloerke et al. 2021) and the software Keynote. The full script is available in the Supporting Information.

## 3 Results

### 3.1 Impact of Skin Tumors on the Sex Ratio (F0 Generation)

Out of the 377 embryos injected with the oncogenic plasmid surviving until the 13th week, 60.6% (*n* = 228) were GFP‐positive 48 h later, indicating that they expressed the H‐RASV12 oncogene. Out of the 77 female fish (see Figure 1) that incorporated the plasmid and survived for 13 weeks, 15 developed tumors and were used as mothers for the next experiment. No statistically significant difference in the sex ratios of fish in the F0 generation was observed between those expressing the oncogenic plasmid (GFP+, Figure 2B, $\hat{p}$ = 0.52 [Confidence interval = 0.4338; 0.60], *n* = 228) and the control group (GFP−, Figure 2B, $\hat{p}$ = 0.48 [0.401; 0.54], *n* = 149), as the best model selected did not include this effect. AICs of different models explaining the sex ratio in the F0 generation are shown in Table S1.

### 3.2 Impact of Skin Tumors on the Survival and Sex Ratio of the Offspring (F1 Generation) at the 13th Week

After mating with wild‐type males, cancerous females (GFP+) produced more embryos (Figure 2C, $\hat{\mu }$ = 159.8 [124.0; 195.6], *n* = 15) compared to control females (GFP−, $\hat{\mu }$ = 135.6 [117.4; 153.8], *n* = 20). The incidence rate ratio (IRR) was 1.26 [1.18–1.34], indicating that, on average, a cancerous female produced 1.26 embryos for every one produced by a non‐cancerous female, with random variations observed across mating dates (see Table S2).

Embryos from cancerous mothers were more likely to survive until their 13th week (Figure 2D, $\hat{\mu }$ = 35.6% [27.8; 43.4], *n* = 15) compared to those born from control mothers (GFP−, $\hat{\mu }$ = 27.5% [23.6; 31.3], *n* = 20). The IRR was 1.30 [1.14–1.48], indicating a 1.30 times higher likelihood for embryos from cancerous mothers to reach maturity by the 13th week, with random variations observed across mating dates too. Residual diagnostics show a slight deviation from uniformity, but with no signs of overdispersion or variance heterogeneity (see Table S3 and Supporting Information S1: Section 3).

Regarding the sex ratio of the F1 generation of fish (Figure 2E), statistical model selection revealed no significant influence of the cancerous state of the mother; the best model selected did not include this effect (see Table S4, *n* = 35).

## 4 Discussion

The impact of oncogenic processes on the evolutionary ecology of wildlife is a rapidly expanding area of research. However, our understanding is currently limited; we are far from having explored the full spectrum of biological traits that could potentially be influenced by malignant progression (Thomas et al. 2017).

Chemical pollution in ecosystems has been shown to influence the sex ratio in wild animal populations, particularly in species whose sex is determined by the environment (Decourten and Brander 2017; Mikó et al. 2021; Miracle et al. 2011). For instance, in the zebrafish, exposure to clotrimazole (an endocrine disrupting chemical) induces male‐skewed sex ratios (Brown et al. 2015). Interpretation of the causes responsible for these skews is often complex because multiple factors may act together, including the modes of actions of chemicals, temperature, inbreeding, or environmental parameters such as stress (Dang 2016; Dang and Kienzler 2019). Thus, the extent to which diseases such as cancer, resulting from pollution, directly impact the sex ratio in populations remains uncertain. Our findings do not support the hypothesis that cancer progression itself is involved in sex ratio variation, neither directly nor through an adaptive response from the host. Indeed, under the conditions of Experiment 1, our observations indicate that cancer does not appear to be associated with differential sex‐specific mortality at the embryonic stage, nor does it play a role in sex determination (since both phenomena would have skewed the sex ratio). These results contrast with numerous studies that have identified sex‐specific biological responses in zebrafish regarding various factors (Giommi et al. 2022; Li et al. 2019; Lin et al. 2022; Park et al. 2022; van Gelderen et al. 2022; Zhai et al. 2022; Zheng et al. 2022; Zhong et al. 2023; Zhou et al. 2010), including cancers (Lee et al. 2021, 2018; Mensah et al. 2019; Montal et al. 2023). One possible explanation is that the cancer model used in our study may have too low aggressiveness, as possibly evidenced by the ability of individuals affected at the embryo stage to develop into adulthood. Indeed, the HRAS G12V plasmid we used has been shown to induce tumors with high penetrance and at an early developmental stage in zebrafish, making it an effective tool for studying the impact of melanoma on sex determination, as these processes occur simultaneously (Santoriello et al. 2009). However, the level of mosaicism in the plasmid's expression may have influenced our results. Approximately 20% of adult fish injected with the plasmid developed neoplasia, while a varying proportion exhibited other forms of hyperpigmentation. The variability in tumor expression and potential differences in tumor aggressiveness could have influenced both sex‐specific survival and reproductive success. As we did not quantify RASV12 overexpression levels in mosaic embryos, we acknowledge this as a limitation of our study. These factors may have mitigated the severity of the cancer's impact on fish reproduction, potentially contributing to the statistically non‐significant differences observed in our study and explaining the contradiction with other research findings. Future studies employing more aggressive cancer models or quantifying mosaicism and expression levels may provide further insights into these dynamics. Another limitation of our study is the incomplete tracking of tumor development in injected females. Among the 77 females that incorporated the plasmid, we observed 15 with tumors; however, this number may underestimate the true prevalence of neoplasia due to potential early mortality before tumors could be identified. This lack of systematic data for the entire cohort highlights the need for more comprehensive monitoring of tumor development across all individuals in future studies. Such tracking would provide a more accurate understanding of the relationship between tumor development, survival, and reproductive traits. In addition, the incomplete documentation of tumor development across all GFP‐positive injected embryos, particularly in males, and in females was not included in subsequent experiments. While previous studies report approximately 20% neoplasia among injected individuals (Santoriello et al. 2009), we could not systematically confirm this proportion in our dataset due to the lack of photographic records or regular checks for neoplasia across the entire cohort. This gap underscores the need for future experiments to implement comprehensive and consistent tracking of tumor development in both sexes to better understand the relationship between GFP expression, mosaicism, and tumor phenotypes.

An alternative explanation for the absence of cancer influence on the sex ratio is that the latter is adjusted in relation to growth. In many fishes, such as Atlantic silversides ( *Menidia menidia* ), larger females produce more eggs than smaller females, while the fecundity of males is less dependent on body size because males do not typically monopolize mating opportunities (Conover and Kynard 1981). Thus, the fitness benefit of larger size is greater for females than for males. Growth‐dependent sex determination may maximize reproduction in zebrafish in the same way, with faster‐growing individuals developing as females and slower‐growing individuals as males (Lawrence et al. 2008). If tumors impede organismal growth, as observed for intestinal tumors (Enya et al. 2018), this should select for sex ratio adjustment in the opposite direction (towards males), to that expected by sex differential embryonic mortality (towards females) (Wells 2000). Thus, the predicted direction of sex ratio adjustment may depend on the relative importance of these two opposing forces and could lead to selection for no sex ratio adjustments. Further work with a larger sample size is needed to follow up on this study, examining the consequences of skin tumors on growth rates and the condition factor of fish. The variability in tumor aggressiveness across different models highlights the importance of choosing cancer models that align with the specific biological questions being addressed. Model organisms with more aggressive or consistently expressed cancers may reveal additional insights into the interplay between cancer progression and reproductive traits. Additionally, it would be interesting to include inter‐individual differences in tumor development in future experiments. Future studies could further investigate the effects of neoplasia on allocation to reproduction by measuring traits such as lifespan, long‐term reproductive patterns, and phenotypic changes in affected individuals. Using alternative models that allow extended observations could provide deeper insights into life‐history trade‐offs and the fitness impacts of the disease.

Considering Experiment 2, our results show that there is no statistically significant difference in the sex ratio of broods from cancerous and non‐cancerous females. Although many fish species now develop cancers in the wild (Baines et al. 2021), no study has explored the links between this pathology and egg or sperm quality. Further studies would be necessary to clarify this point. An intriguing pattern observed in the present study, consistent with prior research on the reproductive biology of organisms bearing tumors (Arnal et al. 2017; Boutry, Mistral, et al. 2022; Jones et al. 2008), is that females with cancer appear to produce more eggs with higher chances to survive to reach sexual maturity than healthy females. This supports the idea of a greater allocation of energy to reproductive resources in females whose life expectancy is compromised by cancer progression. However, further studies on the quality of eggs and embryos produced by cancerous and non‐cancerous females would be necessary to provide additional information on maternal investment in eggs. Further data, particularly on other fish/cancer models, would also be needed to establish whether our findings can be generalized.

In summary, this pioneering study explores the complex responses of an aquatic vertebrate organism to oncogenic processes at different life stages. The influence of skin cancer on the reproductive ecology of zebrafish does not support the hypothesis that disease progression interferes with the mechanisms governing sex‐ratio variations in this species, either directly or through adaptive responses. Nevertheless, the results are consistent with previous studies suggesting that organisms that develop cancer tend to maximize their immediate reproductive effort before dying prematurely. This study highlights the eco‐evolutionary dynamics of the responses developed by organisms increasingly confronted with oncogenic compounds in contemporary ecosystems polluted by anthropogenic activities. However, to deepen our understanding, future studies should prioritize systematic tracking of tumor development in all injected embryos. This will help address current limitations related to the variability in mosaicism and incomplete records of neoplasia, ultimately allowing for more robust conclusions about the interplay between cancer progression and reproductive dynamics. Future studies would also benefit from precise quantification of plasmid expression levels to better understand the impact of mosaicism on experimental outcomes. Additionally, employing more aggressive or uniformly expressed cancer models could help elucidate the interplay between tumor progression and key reproductive traits.

## Author Contributions

**Justine Boutry:** conceptualization (lead), data curation (lead), formal analysis (lead), methodology (supporting), visualization (lead), writing – original draft (equal), writing – review and editing (lead). **Mathieu Douhard:** conceptualization (equal), supervision (equal), writing – review and editing (equal). **Klara Asselin:** conceptualization (supporting), writing – review and editing (supporting). **Antoine M. Dujon:** conceptualization (equal), writing – review and editing (equal). **Jordan Meliani:** conceptualization (equal), writing – review and editing (equal). **Olivier De Backer:** conceptualization (equal), writing – review and editing (equal). **Delphine Nicolas:** conceptualization (equal), writing – review and editing (equal). **Aaron G. Schultz:** conceptualization (equal), writing – review and editing (equal). **Peter A. Biro:** conceptualization (equal), writing – review and editing (equal). **Christa Beckmann:** conceptualization (equal), writing – review and editing (equal). **Laura Fontenille:** conceptualization (equal), data curation (equal), investigation (lead), writing – original draft (equal). **Karima Kissa:** conceptualization (equal), data curation (equal), investigation (equal), methodology (equal), writing – original draft (equal), writing – review and editing (equal). **Beata Ujvari:** conceptualization (equal), writing – review and editing (equal). **Frédéric Thomas:** conceptualization (equal), funding acquisition (equal), investigation (equal), methodology (lead), supervision (equal), writing – original draft (equal), writing – review and editing (equal).

## Conflicts of Interest

The authors declare no conflicts of interest.

## Supporting information


**Data S1:** ece372003‐sup‐0001‐Supinfo.docx.

## Data Availability

All datasets and R scripts used for statistical analysis and figure generation are available in the Supporting Information provided with this submission.
