# Supplementary material for: Consequences of Cancer on Zebrafish Danio rerio: Insights Into Sex Determination, Sex Ratio, and Offspring Survival
Source: Ecol Evol. 2025 Aug 31;15(9):e72003. doi: 10.1002/ece3.72003 (PMC12399573; doi:10.1002/ece3.72003)
Supplement: Supplementary file 1 — Data S1: ece372003‐sup‐0001‐Supinfo.docx. [file ECE3-15-e72003-s001.docx]

SUPPLEMENTARY TABLES

| **Random model selection** | **Df** | **AICc** | **Weights** |
| --- | --- | --- | --- |
| Sex ratio ~ GFP * Total number of individuals + *(1\|Exp/Aquarium)* | 6 | 87.38 | 0.009 |
| Sex ratio ~ GFP * Total number of individuals + *(1\|Aquarium)* | 5 | 82.43 | 0.112 |
| Sex ratio ~ GFP * Total number of individuals | 4 | 78.32 | 0.879 |
| **Fixed model selection** | **Df** | **AICc** | **Weights** |
| Sex ratio ~ GFP * Total number of individuals | 2 | 91.39 | 0.000 |
| Sex ratio ~ GFP + Total number of individuals | 4 | 85.56 | 0.003 |
| Sex ratio ~ GFP | 3 | 77.84 | 0.121 |
| Sex ratio ~ Total number of individuals | 3 | 81.90 | 0.016 |
| Sex ratio ~ 1 | 5 | 73.91 | 0.864 |

**Table S1:** Results from binomial generalized linear (mixed) models with the sex ratio in the F0 generation of zebrafish (*Danio rerio*) as response variable. Models with variations in their random or fixed effect are compared using degrees of freedom, AICc values and weights. Random effects are written in italic.

| **Random model selection** | **Df** | **AICc** | **Weights** |
| --- | --- | --- | --- |
| Embryo ~ GFP + *(1\|Exp/Mating date)* | 4 | 647.67 | 0.218 |
| Embryo ~ GFP + *(1\|Mating date)* | 3 | 645.12 | 0.782 |
| Embryo ~ GFP | 2 | 786.12 | 0.000 |
| **Fixed model selection** | **Df** | **AICc** | **Weights** |
| Embryo ~ GFP + *(1\|Mating date)* | 2 | 653.53 | 1.000 |
| Embryo ~ 1 + *(1\|Mating date)* | 3 | 698.04 | 0.000 |
| **Final best model** | **IRR** | **CI** | **P-Value** |
| GFP [pos] | 1.26 | 1.18 – 1.34 | <0.001 |
| Random Effects |  | | |
| σ^2^ | 0.01 | | |
| Nb mating date | 11 | | |
| Marginal R2 / Conditional R2 | 0.186 / 0.905 | | |

**Table S2**: Results from Poisson generalized linear mixed models with the number of embryos (3 days post fertilization) in the F1 generation of zebrafish (*Danio rerio*) as the response variable. Models with variations in their random or fixed effect are compared using degrees of freedom, AICc values and weights. The estimator of the fixed and random effect of the best model is shown in the last part of the table, with their associated incidence rate ratio (IRR), confidence interval (CI), p-value, number of random categories, residual variance (σ^2^), marginal and conditional correlation coefficient (R^2^). Random effects are written in italic.

| **Random model selection** | **Df** | **AICc** | **Weights** |
| --- | --- | --- | --- |
| Mature fish / Embryo ~ GFP + *(1\|Exp/Mating date)* | 4 | 454.2 | 0.218 |
| Mature fish / Embryo ~ GFP + *(1\|Mating date)* | 3 | 451.6 | 0.782 |
| Mature fish /Embryo ~ GFP | 2 | 476.1 | 0.000 |
| **Fixed model selection** | **Df** | **AICc** | **Weights** |
| Mature fish / Embryo ~ GFP + *(1\|Mating date)* | 4 | 458.6 | 0.989 |
| Mature fish / Embryo ~ 1 *+ (1\|Mating date)* | 3 | 467.6 | 0.011 |
| **Final best model** | **IRR** | **CI** | **P-Value** |
| GFP [pos] | 1.30 | 1.14 – 1.48 | <0.001 |
| Random Effects |  | | |
| σ^2^ | 0.02 | | |
| Nb mating date | 11 | | |
| Marginal R2 / Conditional R2 | 0.230 / 0.702 | | |

**Table S3**: Results from binomial generalized linear mixed models with the number of mature fish over the number of embryos in the F1 generation of zebrafish (*Danio rerio*) as response variable. Models with variations in their random or fixed effect are compared using degrees of freedom, AICc values and weights. The estimator of the fixed and random effect of the best model is shown in the last part of the table, with their associated incidence rate ratio (IRR), confidence interval (CI), p-value, number of categories, residual variance (σ^2^), marginal and conditional correlation coefficient (R^2^). Random effects are written in italic.

| **Random model selection** | **Df** | **AICc** | **Weights** |
| --- | --- | --- | --- |
| Sex ratio ~ GFP + Nb Mature fish + Embryo + *(1\|Exp/Mating date)* | 6 | 193.06 | 0.045 |
| Sex ratio ~ GFP + Nb Mature fish + Embryo + *(1\|Mating date)* | 5 | 190.12 | 0.194 |
| **Sex ratio ~ GFP + Nb Mature fish + Embryo GFP** | **4** | **187.39** | **0.761** |
| **Fixed model selection** | **Df** | **AICc** | **Weights** |
| Sex ratio ~ GFP x Nb Mature fish x Embryo GFP | 8 | 281.23 | 0.000 |
| Sex ratio ~ GFP + Nb Mature fish x Embryo GFP | 5 | 238.13 | 0.000 |
| Sex ratio ~ GFP x Nb Mature fish + Embryo GFP | 5 | 227.75 | 0.000 |
| Sex ratio ~ GFP x Embryo GFP + Nb Mature fish | 5 | 229.54 | 0.000 |
| Sex ratio ~ Nb Mature fish x Embryo GFP | 4 | 232.58 | 0.000 |
| Sex ratio ~ GFP x Nb Mature fish | 4 | 212.94 | 0.000 |
| Sex ratio ~ GFP x Embryo GFP | 4 | 217.95 | 0.000 |
| Sex ratio ~ Nb Mature fish + Embryo GFP | 3 | 211.40 | 0.000 |
| Sex ratio ~ GFP + Nb Mature fish | 3 | 202.05 | 0.000 |
| Sex ratio ~ GFP + Embryo GFP | 3 | 204.98 | 0.000 |
| Sex ratio ~ GFP + Nb Mature fish + Embryo GFP | 4 | 216.77 | 0.000 |
| Sex ratio ~ Nb embryo GFP | 2 | 199.89 | 0.001 |
| Sex ratio ~ GFP | 2 | 190.49 | 0.082 |
| Sex ratio ~ Nb Mature fish | 2 | 196.84 | 0.003 |
| **Sex ratio ~ 1** | **1** | **185.66** | **0.913** |

**Table S4**: Comparisons of the results of different binomial generalized models explaining the sex-ratio in the F1 generation (binomial distribution) of zebrafish (*Danio rerio*). The degree of freedom, AICc value and weight of each model are shown, random effects are written in italic.
